# Enhanced production of phomapyrrolidone a through culture-condition optimization and morphological regulation in *Didymella* sp. FATR0054

**DOI:** 10.1186/s40643-026-01132-2

**Published:** 2026-09-21

**Authors:** Weiyan Zhou, Zhongyuan Chen, Jiacheng Guo, Yiming Yang, Fan Yang, Xiujuan Xin, Liwei Zhuang, Lizhi Gong, Faliang An

**Affiliations:** 1 State Key Laboratory of Bioreactor Engineering, East China University of Science and Technology, 130 Mei Long Road, Shanghai, 200237 China; 2 School of Chemical Engineering, East China University of Science and Technology, Shanghai, 200237 China; 3 Marine Biomedical Science and Technology Innovation Platform of Lin-Gang Special Area, No. 4, Lane 218, Haiji Sixth Road, Shanghai, 201306 China; 4 Department of Pharmacy, Renji Hospital, School of Medicine, Shanghai Jiao Tong University, Shanghai, 200127 China

**Keywords:** Phomapyrrolidone A, Marine filamentous fungus, Submerged fermentation

## Abstract

Phomapyrrolidone A (Ppd A) is a hirsutellone-type macrocyclic alkaloid derived from marine fungi, exhibiting significant activity against triple-negative breast cancer (TNBC). However, its low fermentation yield has limited further drug development. This study established a systematic optimization strategy integrating medium optimization, mycelial morphology regulation, and computational fluid dynamics (CFD) to enhance Ppd A production by the marine-derived filamentous fungus *Didymella* sp. FATR0054. Medium composition and fermentation parameters were optimized, increasing shake-flask production from 4.52 mg/L to 289.36 mg/L. The process was successfully scaled up in a 5-L bioreactor. Exogenous co-supplementation further increased production by 26% to 365 mg/L. Morphological regulation proved critical for productivity. Mechanical pre-grinding of the seed culture reduced mycelial pellet diameter to approximately 1 mm, creating compact and uniform pellets. This increased Ppd A production to 389.25 mg/L, representing an 86-fold improvement. CFD analysis of the interactions among the flow field, fungal pellet diameter distribution, and product formation identified 450 rpm as a candidate operating compromise for the current 5-L bioreactor, based on the simulated spatial uniformity of the Ppd A concentration field, local energy dissipation, and pellet fragmentation. This study establishes an integrated fermentation intensification strategy combining experimental optimization and flow-field modeling for large-scale production of Ppd A and process regulation in filamentous fungal fermentation.

**Graphical abstract:**

**Figure:**
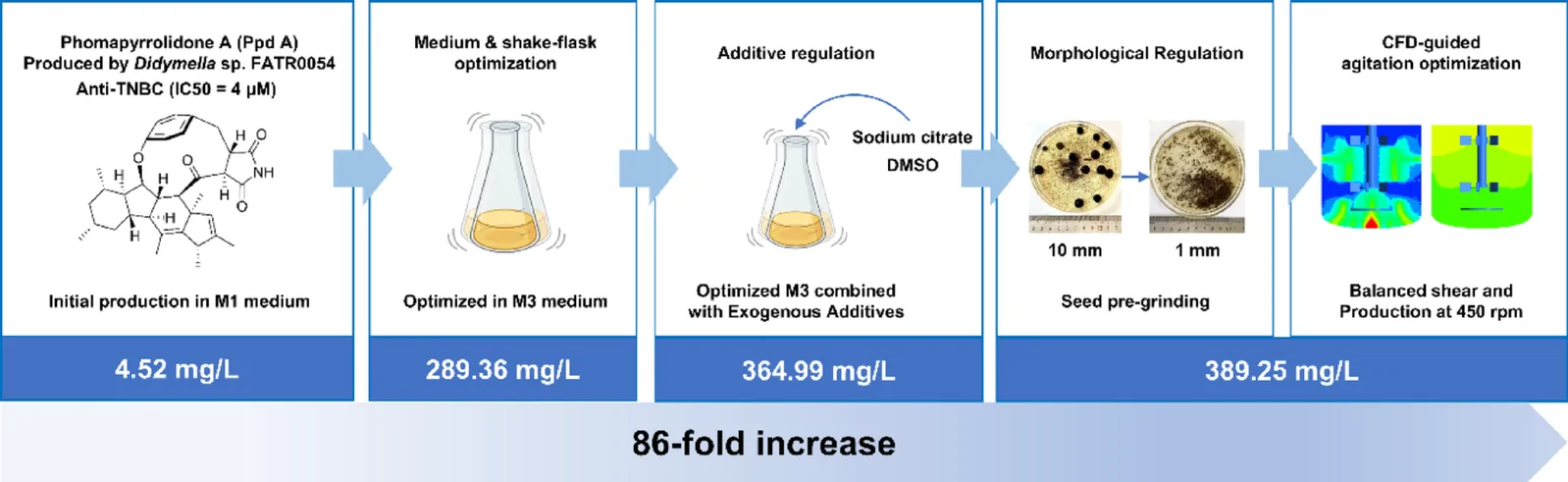

**Supplementary Information:**

The online version contains supplementary material available at https://doi.org/10.1186/s40643-026-01132-2.

## Introduction

Phomapyrrolidone A (Ppd A) is a typical member of the hirsutellone-type alkaloids, a class of polyketide-nonribosomal peptide hybrid-type macrocyclic alkaloids characterized by their thirteen-membered ring structures and intriguing antitumor activities (Li et al. 2013; Ding et al. 2023; Isaka 2007). It was isolated from various fungal genera including *Phoma* (Wijeratne et al. 2013), *Didymella* (Chen et al. 2019), *Terminalia* (Ariantari et al. 2020), *Microascus* (Yao et al. 2024) and *Sarocladium* (Al Subeh et al. 2023). In addition, it has demonstrated potent activity against triple-negative breast cancer (TNBC) along with favorable biosafety profiles (Wijeratne et al. 2013). However, the rarity of its natural sources and extremely low yields from both chemical synthesis and fermentation have limited both pharmacological studies and potential industrial development (Luo et al. 2024; Li et al. 2024). Marine-derived fungi, inhabiting extreme environments such as high salinity, high pressure, and low temperature, have evolved unique metabolic capabilities, enabling the production of structurally novel and bioactive secondary metabolites (Böl et al. 2020; Yang et al. 2025). In our previous work, the marine-derived fungus *Didymella* sp. FATR0054 was screened and identified as a producer of Ppd A under laboratory condition. As a wild-type strain, it has not been subjected to systematic fermentation process development or metabolic regulation studies, and its production under laboratory conditions is insufficient to support subsequent drug development. Ppd A is synthesized in *Didymella* sp. FATR0054 via a PKS-NRPS hybrid pathway, with its yield influenced by multiple factors, including mycelial morphology (Xu et al. 2026; Chu et al. 2025), regulation of biosynthetic pathways (Zhou et al. 2025; Yuan et al. 2025), and fermentation process parameters (Betiku et al. 2025; Bellaouchi et al. 2024).

During submerged liquid fermentation, filamentous fungi generally exhibit two typical morphologies: dispersed hyphae and compact pellets (Xu et al. 2026). The formation of these morphologies is jointly regulated by multiple factors, including physical factors (such as metal ions (Kumar and Dubey 2017), surfactants (Meng et al. 2021), talc powder (Yatmaz et al. 2016), and hydrodynamic conditions (Venkatachalam et al. 2023)), genetic factors (such as *flbE* (Chen et al. 2026) and Rho GTPases (Jiang et al. 2024)), and microbial interactions (such as co-cultivation (Sun et al. 2023)). Different morphologies have significant effects on fermentation performance. In general, dispersed mycelial morphology is more favorable for enzyme production, whereas pellet morphology is more beneficial for the accumulation of secondary metabolites (Xu et al. 2026). This is mainly because highly branched and dispersed hyphae maximize tip density and increase the surface area for enzyme secretion (Adsul et al. 2022), while dispersed mycelia also markedly increase broth viscosity, thereby limiting the transfer and diffusion of oxygen and nutrients and reducing mass transfer efficiency (Cerrone and E. O’Connor 2025). In contrast, pellet morphology facilitates the transport of oxygen and nutrients into the biomass, thereby affecting fungal growth and metabolic states and ultimately enhancing product formation (Saberi et al. 2020). However, larger pellets are not always advantageous. During fermentation, pellets are continuously subjected to turbulence and shear stress and remain in a dynamic balance between growth and fragmentation (Lee et al. 2017; Park et al. 1999). When pellet diameter exceeds a critical size, hollow pellets are likely to form, leading to oxygen-limited regions inside the pellets and causing growth arrest or even death of the core hyphae (Xu et al. 2026). Conversely, excessive shear stress can result in severe fragmentation of the biomass and the loss of normal growth and metabolic activity. Comparatively, small pellets of appropriate size are more favorable for uniform oxygen diffusion into the pellet core, maintaining homogeneous metabolic activity and aerobic conditions while alleviating nutrient limitation, thus promoting the efficient production of oxygen-dependent secondary metabolites (Cerrone and O’Connor 2025).

To systematically investigate the effects of hydrodynamic conditions on pellet growth and fragmentation behavior in stirred bioreactors, computational fluid dynamics (CFD) provides an important approach for analyzing the microscopic flow environment (Moilanen et al. 2006). As a predictive and visualizable tool, CFD can be used to analyze flow field distribution, shear stress, and substrate transport and distribution within bioreactors, and further evaluate their effects on microbial growth, morphological evolution, and metabolite production (Nadal-Rey et al. 2022).

This study aimed to achieve efficient biosynthesis of Ppd A by establishing a multi-level fermentation enhancement strategy involving medium optimization, fermentation process regulation, morphological control, and bioreactor hydrodynamic optimization. First, the nutritional composition of the medium was optimized using Plackett–Burman (PB) design and response surface methodology, followed by the determination of optimal shake-flask fermentation conditions. Subsequently, the addition of biosynthetic precursors, metal ions, and surfactants was applied to further improve Ppd A production. On this basis, the effects of different nitrogen sources and physical shear conditions on fungal morphology and product biosynthesis were investigated to identify morphological characteristics favorable for Ppd A production. Finally, a CFD-PBM model was employed to compare the flow fields, pellet size distributions, and product formation trends in a 5-L bioreactor under different agitation speeds. This study not only deepens the understanding of the biosynthetic regulation of polyketide macrocyclic alkaloids, but also provides theoretical and practical guidance for the efficient development and fermentation optimization of PKS-NRPS-derived marine natural products.

## Materials and methods

### Strain and culture media

#### Strain

The fungal strain *D*. sp. FATR0054 was isolated from a sea crab gathered from the intertidal zone of Zhoushan, Zhejiang, China. Identification of the fungal strain was accomplished through an examination of its morphological attributes and sequencing of the fungal Internal Transcribed Spacer (ITS) region in ribosomal DNA (rDNA). The fungal strain is preserved in the laboratory of the State Key Laboratory of Bioreactor Engineering in Shanghai at a temperature of − 80 °C.

Strain preservation: The strain was cultured on agar plates for approximately 7 days until abundant sporulation was observed. The surface spores were gently scraped with a sterile flat-tipped toothpick, suspended in a 40% (v/v) aqueous glycerol solution, and stored at − 80 °C.

#### Culture media

Solid medium (M1-S): Glucose 30 g/L, peptone 10 g/L, yeast extract 2 g/L, KH_2_PO_4_ 1 g/L, MgSO_4_·7H_2_O 0.5 g/L, and agar 20 g/L.

Liquid seed medium (M1-L): Glucose 30 g/L, peptone 10 g/L, yeast extract 2 g/L, KH_2_PO_4_ 1 g/L, MgSO_4_·7H_2_O 0.5 g/L.

Initial fermentation medium: The detailed compositions of the basal media (M1-M11) used for preliminary screening are provided in the Supplementary Materials Table S2.

Optimized fermentation medium: maltose 35 g/L, yeast extract powder 12.5 g/L, FeSO_4_∙7H_2_O 0.00618 g/L, MgSO_4_∙7H_2_O 0.42 g/L, NaCl 0.625 g/L, KH_2_PO_4_ 1.25 g/L, and NaNO_3_ 2.25 g/L, pH = 7.

Detailed information on reagents and instruments is provided in the Supplementary Materials Tables S3 and S4.

### Initial culture conditions

Plate cultivation: Spore suspensions of the *Didymella* sp. FATR0054 stored at − 80 °C were thawed and inoculated onto M1-S medium using sterile toothpicks. The plates were incubated in an inverted position at 28 °C for 5–7 days.

Seed culture preparation: Agar plugs (approximately 1 cm^2^) excised from colonies on M1-S plate were inoculated into 250 mL flasks containing 100 mL of M1-L medium and incubated at 28 °C and 200 r/min on a rotary shaker for 72 h.

Initial shake flask fermentation: The seed culture grown in M1-L medium for 72 h was inoculated at 5% (v/v) into 250 mL shake flasks containing 100 mL of fermentation medium and cultivated at 28 °C and 200 r/min for 168 h.

Optimized shake flask fermentation: The seed culture grown in M1-L medium for 72 h was inoculated at 5% (v/v) into 250 mL shake flasks containing 100 mL of optimized fermentation medium in “Culture media” Section and cultivated at 28 °C and 200 r/min for 168 h.

5-L bioreactor cultivation: Fermentation was carried out in a 5-L stirred tank bioreactor (Shanghai Guoqiang Biochemical Engineering Equipment Co., Ltd) equipped with dual Rushton turbine impellers at a working volume of 3 L. The medium composition was as optimized in “Culture media” Section. The cultivation temperature was maintained at 28 °C with an inoculum size of 5% (v/v). Agitation speed and aeration rate were regulated through a dissolved oxygen (DO)-cascaded control strategy to ensure sufficient oxygen supply for fungal growth.

### Fermentation sample processing

#### Determination of dry cell weight

Fermentation broth was separated by vacuum filtration, and the collected mycelia were retained on pre-weighed qualitative filter paper. The residues were washed three times with deionized water to remove impurities and then dried to a constant weight in a vacuum oven at 50 °C. After cooling, the dry weight was accurately recorded.

#### Ppd A extraction

One hundred milliliters of fermentation broth were filtered under vacuum to separate the mycelia from the supernatant. The collected mycelia were immersed in 50 mL methanol and disrupted using ultrasonication. The resulting extract was concentrated under reduced pressure at 50 °C using a rotary evaporator and dried. The residue was redissolved in 10 mL of methanol, and 1.5 mL of the solution was transferred into a 2.0 mL microcentrifuge tube and centrifuged at 13,000 rpm to remove insoluble impurities. The supernatant was filtered through a 0.22 μm membrane into an HPLC vial and stored at 4 °C until analysis. The Ppd A content in the mycelial extract was quantified using HPLC with an external standard method.

#### Analysis of Ppd A by HPLC

Ppd A was analyzed by high-performance liquid chromatography (HPLC) using an Agilent ZORBAX Eclipse SB-CN column. The mobile phase consisted of water (A) and methanol (B) with the following gradient: 0 min, 90% methanol; 15 min, 100% methanol; 26 min, 90% methanol; total runtime of 30 min. The flow rate was 1.0 mL/min, the column temperature was maintained at 25 °C, and detection was performed at 210 nm.

### RNA extraction and transcript quantification

Biomass samples were collected at 168 h of fermentation to characterize the transcriptional status of the biosynthetic gene cluster during the late stage of production. 1 mL of fermentation broth was transferred into a 1.5 mL microcentrifuge tube and centrifuged at 12,000 rpm for 5 min. The supernatant was discarded, and the pellet was washed three times with PBS buffer, with thorough resuspension after each wash to remove residual fermentation medium. The washed mycelia were stored at − 80 °C until RNA extraction, which was performed using the NCM Biotech RNA kit according to the manufacturer’s instructions.

RNA concentrations were measured using a NanoDrop 2000 spectrophotometer. For each sample, 1 μg of total RNA was treated to remove genomic DNA and reverse-transcribed into cDNA using the Hifair III 1st Strand cDNA Synthesis Kit (gDNA digester plus). Transcriptional levels were determined using Hieff UNICON Advanced qPCR SYBR Master Mix on a CFX96™ Real-Time PCR Detection System. *GAPDH* was used as the reference gene. Three biological replicates and four technical replicates were performed, and relative transcriptional levels were calculated using the 2^−∆∆Ct^ method. The primer sequences and reaction system for RT-qPCR used in this study are listed in Supporting Materials Tables S5 and S6.

### CFD numerical simulation

The simulations were conducted using ANSYS Fluent 2021R1. Given the relatively low apparent viscosity of the culture system, water was simplified to a continuous phase in the single-phase steady-state simulations. The Realizable k-ε turbulence model was employed, and impeller rotation was treated using the Multiple Reference Frame (MRF) approach. The SIMPLE algorithm was used for pressure–velocity coupling, and the average flow velocity and turbulent energy dissipation rate were monitored. Once residuals converged and monitored parameters stabilized, an Euler-Euler multiphase flow model was applied. A second phase representing the fungal biomass, with a density of 1200 kg/m^3^, was introduced, and the Population Balance Model (PBM) was implemented to simulate fungal pellet growth and breakage.

Pellet size classifications were set at 0.5, 1.5, 2.5, 3.5, 4.5 and 5.5 mm. User-Defined Functions (UDFs) were employed to input pellet growth rates and turbulence-driven breakage rates into the Growth Rate model and Breakage Kernel, respectively. During initialization, a spherical region of 1.5 mm diameter was patched as the initial fungal distribution, with a volume fraction of 0.9 for 0.5 mm pellets, 0.074 for 1.5 mm pellets, and 0.026 for 2.5 mm pellets.

Transient simulations were then performed. The first 10 s simulated the convective distribution of the fungal phase by the flow field. Subsequently, a product formation source term was introduced using a user-defined scalar (UDS). The simulation was continued for 20 s with a time step of 0.01 s, resulting in a total transient simulation time of 30 s. Ppd A formation followed partially growth-associated kinetics, and the corresponding kinetic equations and parameters are provided in the Supplementary Materials.

## Results

**Fig. 1:**
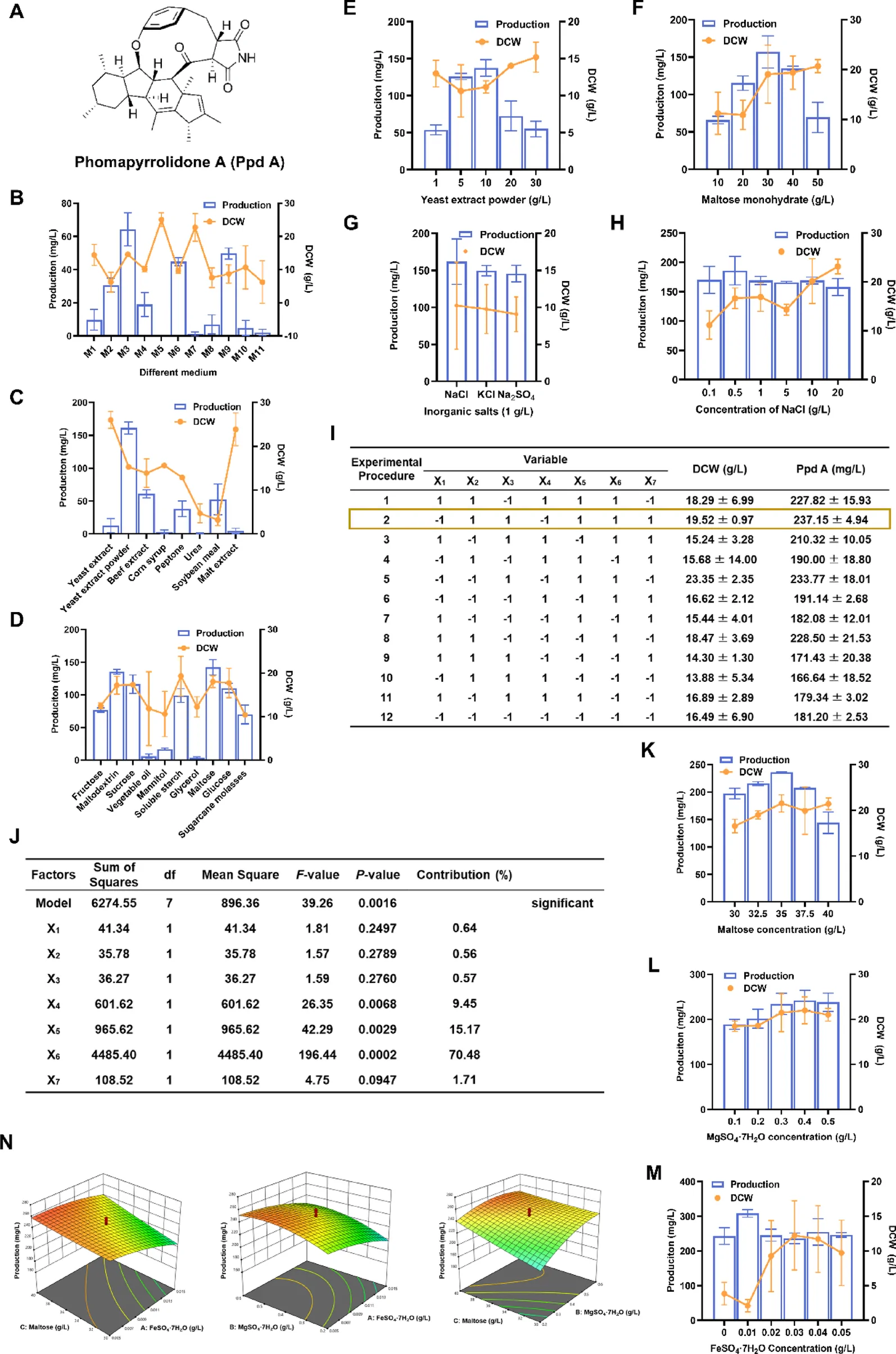
Statistical optimization of fermentation medium for enhanced Ppd A production **A** Chemical structure of Ppd A. **B** Screening of basal culture media for Ppd A production. **C** Single-factor optimization of nitrogen sources. **D** Single-factor optimization of carbon sources. **E** Screening of yeast extract powder concentration for supplementation. **F** Screening of maltose monohydrate concentration for supplementation. **G** Screening of salt concentration for Ppd A production. **H** Screening of NaCl concentration for supplementation. **I** Plackett–Burman design-based screening and analysis of significant medium components. **J** Statistical evaluation of the significance of individual factors affecting Ppd A production. **K** Effects of concentration gradients of maltose on Ppd A yield. **L** Effects of concentration gradients of MgSO_4_∙7H_2_O on Ppd A yield. **M** Effects of concentration gradients of FeSO_4_∙7H_2_O on Ppd A yield. **N** Three-dimensional response surface plots illustrating the interactions among three key factors and their effects on Ppd A production

In our previous work, Ppd A was isolated from the secondary metabolites of the marine-derived fungus *Didymella* sp. FATR0054 (Gong et al. 2026). The chemical structure of Ppd A is shown in Fig. 1A. However, the production of Ppd A in the original medium M1 was less than 10 mg/L. To improve the detection accuracy of Ppd A, meet the requirements for subsequent drug development, and investigate the influence of fungal morphology on product yield, we first carried out a systematic optimization of the fermentation medium composition and process parameters.

### High-yield medium development

#### Screening of base media and single-factor optimization

In Fig. 1B, a series of basal media (M1–M11) were evaluated. Among them, medium M3 yielded the highest Ppd A production, reaching 62.24 mg/L, which was 6.58-fold higher than that obtained in the initial medium M1. Based on this result, the carbon and nitrogen sources in M3 were replaced for further single-factor optimization. During nitrogen source screening (Fig. 1C), yeast extract powder resulted in the highest Ppd A production. Although yeast extract and malt extract supported higher biomass, the yields of Ppd A were relatively low. In carbon source screening (Fig. 1D), maltose, maltodextrin, sucrose, and glucose all promoted high Ppd A production, with maltose showing the most favorable effect and simultaneously supporting higher dry cell weight. Therefore, maltose and yeast extract powder were selected as the optimal carbon and nitrogen sources, respectively. Notably, during carbon source screening, a good correlation was observed between dry cell weight and Ppd A yield, whereas during nitrogen source screening, the highest yield did not necessarily correspond to the highest biomass, providing a basis for further investigation into the effect of nitrogen sources on fungal morphology.

As shown in Fig. 1E and F, the concentrations of yeast extract powder and maltose were further optimized. With increasing yeast extract powder concentration, Ppd A production first increased and then decreased, reaching a maximum at 10 g/L, while the dry cell weight initially decreased and then increased. Optimization of maltose concentration revealed a similar trend: Ppd A production increased and then declined with increasing maltose concentration, reaching a maximum at 30 g/L. In contrast, dry cell weight increased continuously with increasing maltose concentration, indicating that higher carbon levels promoted biomass growth. Although elevated carbon concentrations enhanced cell growth, excessive carbon supply may negatively affect secondary metabolite biosynthesis. A balance between product formation and biomass accumulation is required. Under the optimized concentrations of 10 g/L yeast extract powder and 30 g/L maltose, Ppd A production reached 156.88 mg/L, representing a 2.52-fold increase compared with that in medium M3.

Considering that marine fungi are adapted to high-salinity environments, inorganic salt screening and concentration optimization were also performed (Fig. 1G and H). The results showed that Ppd A production reached the highest level of 185.70 mg/L at a NaCl concentration of 0.5 g/L. NaCl plays an important role in regulating the metabolism of marine-derived organisms, and appropriate levels of Na^+^ and Cl^−^ may contribute to maintaining cellular osmotic balance and stabilizing metabolic pathway expression.

#### Optimization of culture medium composition using statistical methods

To obtain a high-yield fermentation medium, a Plackett–Burman (PB) experimental design was employed to screen key factors affecting Ppd A production (Fig. 1I). Based on previous single-factor optimization results, seven variables were selected as major factors, including NaNO_3_ (X1), KH_2_PO_4_ (X2), NaCl (X3), MgSO_4_∙7H_2_O (X4), FeSO_4_∙7H_2_O (X5), maltose (X6), and yeast extract powder (X7). The Ppd A yields obtained under different experimental combinations ranged from 166.64 to 237.15 mg/L, indicating significant differences among groups. Analysis of variance (ANOVA) and multiple linear regression analysis were performed to evaluate the significance of each factor and to construct a predictive model (Fig. 1J). The coefficient of determination (R^2^) of the model was 0.9857, and the *P* value was 0.0016, indicating that the regression model exhibited good fitting accuracy and predictive capability. Among the seven tested factors, maltose, FeSO_4_∙7H_2_O, and MgSO_4_∙7H_2_O showed significant effects on Ppd A production (*P* < 0.05), with contribution rates of 70.48%, 15.17%, and 9.45%, respectively. Maltose exhibited the highest contribution. As a disaccharide composed of two glucose units, maltose is hydrolyzed more slowly than glucose, which may reduce carbon catabolite repression caused by rapid glucose metabolism and thereby favor secondary metabolite biosynthesis.

Subsequently, single-factor concentration gradient experiments were conducted to determine the appropriate ranges of the three significant factors. As shown in Fig. 1K–M, the center points were determined to be 35 g/L maltose, 0.4 g/L MgSO_4_∙7H_2_O, and 0.01 g/L FeSO_4_∙7H_2_O. To further determine the optimal response region for Ppd A production, a central composite design (CCD) involving three factors at five levels was carried out, using maltose, FeSO_4_∙7H_2_O, and MgSO_4_∙7H_2_O as independent variables and Ppd A production as the response variable. (The CCD matrix and ANOVA results are provided in the Supplementary Materials Tables S7 and S8.) The ANOVA results showed that the regression model was significant (*P* = 0.0007). The model exhibited good fitting performance (R^2^ = 0.8965), indicating that it was suitable for analyzing factor interactions and predicting optimal combinations. The three-dimensional response surface plots are shown in Fig. 1N. The optimal conditions predicted by Design-Expert software were 35 g/L maltose, 0.42 g/L MgSO_4_∙7H_2_O, and 0.00618 g/L FeSO_4_∙7H_2_O, with a predicted Ppd A yield of 258.53 mg/L. To validate the reliability of the model, three independent parallel fermentation batches were conducted in shake flasks, each with three replicates. The measured Ppd A yields were 264.69 ± 11.98 mg/L, 253.33 ± 14.18 mg/L, and 248.86 ± 7.62 mg/L, with an average yield of 255.63 mg/L. The deviation between the predicted value and the experimental mean was 1.13%, indicating that the model possessed good accuracy and reliability.

Through statistical optimization of medium components, the optimized medium was as follows: maltose 35 g/L, yeast extract powder 12.5 g/L, FeSO_4_∙7H_2_O 0.00618 g/L, MgSO_4_∙7H_2_O 0.42 g/L, NaCl 0.625 g/L, KH_2_PO_4_ 1.25 g/L, NaNO_3_ 2.25 g/L. Ppd A production was increased to 4.11-fold of that obtained in medium M3.

### Optimization of fermentation parameters

**Fig. 2:**
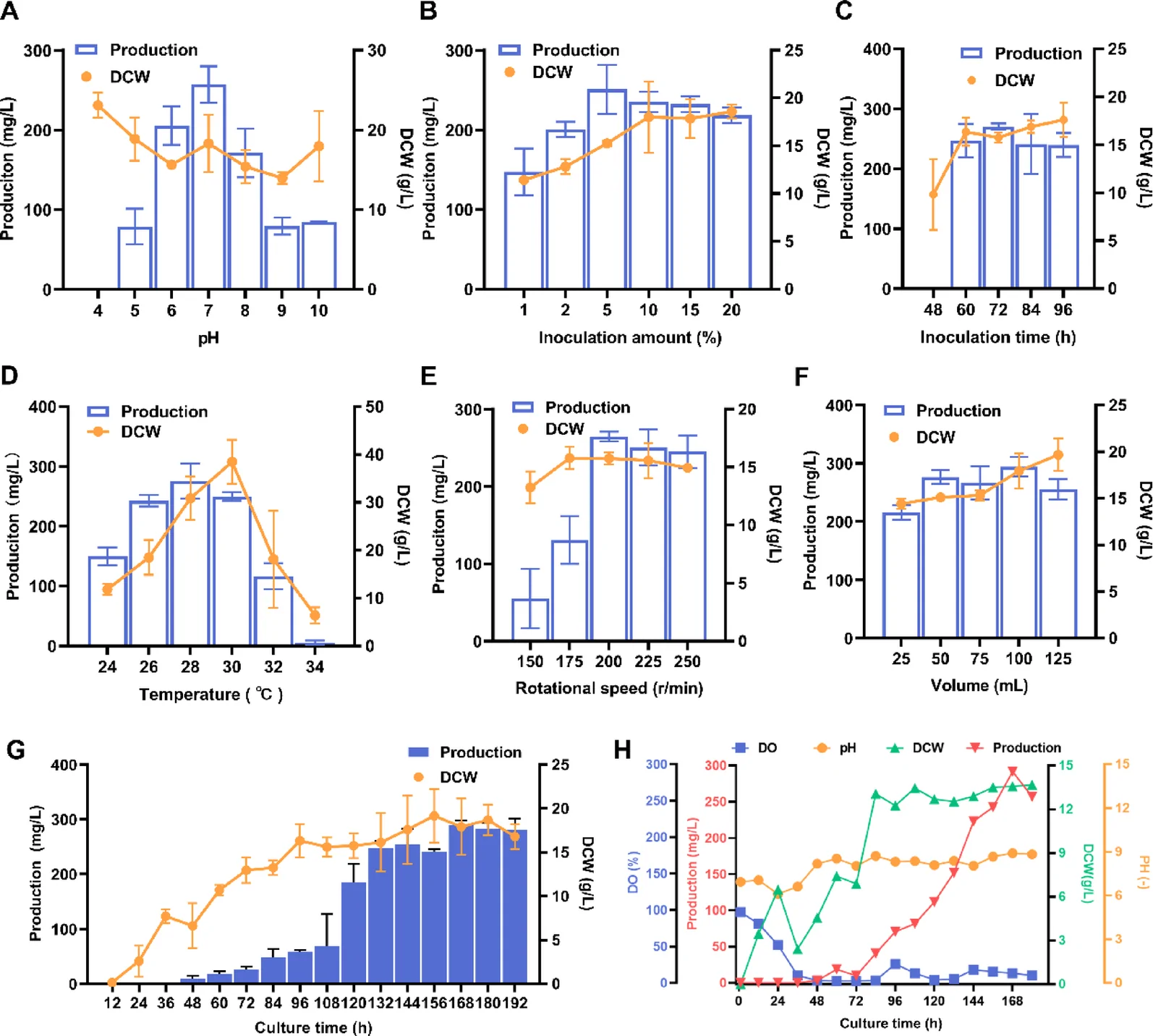
Optimization of fermentation process parameters and scale-up validation in a 5-L bioreactor. Optimization of key fermentation process parameters [**A** pH; **B** Inoculation amount; **C** Inoculation time; **D** Temperature; **E** Rotational speed; **F** Volume] in shake-flask cultures for Ppd A production. **G** Shake-flask fermentation profile; **H** Scale-up of Ppd A fermentation in a 5-L bioreactor: time profiles of Ppd A production and dry cell weight, and corresponding bioreactor operating parameters during the fermentation process

To further improve Ppd A production, key fermentation process parameters in shake-flask culture were optimized, including initial pH, inoculum size, seed age, cultivation temperature, agitation speed, and working volume (Fig. 2A–F). When pH was 7, inoculum size was 5% (v/v) and seed age was 72 h, the highest Ppd A yield (of 270.42 mg/L) was obtained. Regarding temperature, 28 °C was found to be the most suitable for both product formation and biomass accumulation. When the agitation speed exceeded 200 rpm, improved dissolved oxygen transfer promoted fungal growth while maintaining appropriate pellet size, thereby preventing the formation of excessively large pellets and alleviating internal mass transfer limitations. In 250-mL shake flasks, increasing the volume from 25 to 100 mL gradually enhanced Ppd A production, with the highest yield observed at 100 mL.

Based on these results, the optimized fermentation conditions were determined as follows: the initial medium pH was adjusted to 7 before sterilization; 5 mL of 72-h seed culture was inoculated into 100 mL of medium in a 250-mL flask; cultivation was carried out at 28 °C and 200 rpm for 168 h. Under these optimized conditions, Ppd A production reached 289.36 mg/L (Fig. 2G), representing a 13.19% increase compared with the pre-optimization level, and the dry cell weight reached 17.91 g/L.

Subsequently, scale-up fermentation was performed in a 5-L bioreactor (Fig. 2H). After 7d fermentation, Ppd A production reached 291.75 mg/L, demonstrating that the optimized process was stable and reproducible upon scale-up.

### Optimization of Ppd A production via exogenous additives

To further enhance the production of the alkaloid Ppd A by the marine-derived fungus *Didymella* sp. FATR0054, various biosynthesis-related exogenous compounds were supplemented to systematically evaluate their effects on product yield and transcriptional regulation of the biosynthetic gene cluster.

#### Addition of biosynthetic precursors

**Fig. 3:**
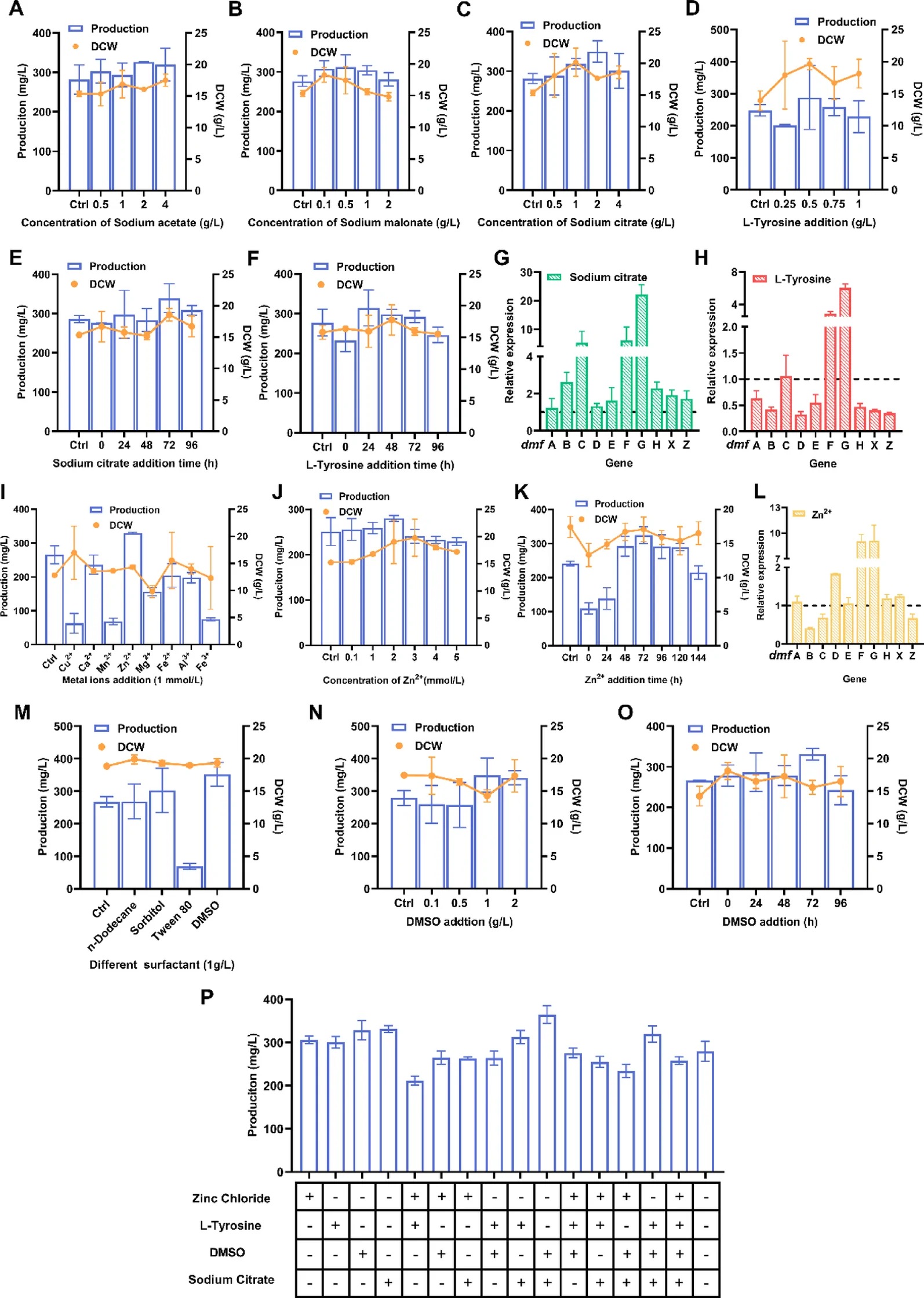
Enhancement of Ppd A production through exogenous supplementation strategies. Screening of supplementation concentrations of biosynthetic precursors related to Ppd A production, including sodium acetate (**A**), sodium malonate (**B**), sodium citrate (**C**), and L-tyrosine (**D**). Optimization of supplementation time for sodium citrate (**E**) and L-tyrosine (**F**). **G** Transcription levels of the Ppd A biosynthetic gene cluster after supplementation with 2 g/L sodium citrate at 72 h. **H** Transcription levels of the Ppd A biosynthetic gene cluster after supplementation with 0.5 g/L L-tyrosine at 24 h. **I** Screening of metal ion supplementation. Optimization of Zn^2+^ supplementation concentration (**J**) and supplementation time (**K**). **L** Transcription levels of the Ppd A biosynthetic gene cluster after supplementation with 2 mmol/L zinc ions at 72 h. **M** Screening of surfactants for their effects on Ppd A production. Optimization of DMSO supplementation concentration (**N**) and addition timing (**O**) for Ppd A production. **P** Evaluation of the combined supplementation with sodium citrate, L-tyrosine, Zn^2+^, and DMSO for improving Ppd A production

Ppd A is a PKS-NRPS hybrid alkaloid composed of a tyrosine-derived moiety and a polyketide chain. We first evaluated the effects of adding sodium acetate (a precursor of acetyl-CoA), sodium malonate (a precursor of malonyl-CoA), sodium citrate (a key intermediate of TCA cycle), and L-tyrosine (a structural precursor incorporated by NRPS) (Fig. 3A–D). The highest Ppd A titer was obtained upon adding 2 g/L sodium acetate at 326.56 mg/L, an approximately 15.9% increase relative to the control. When 0.5 g/L sodium malonate was added, Ppd A production reached 312.20 mg/L, corresponding to an 11.5% increase. The addition of 2 g/L sodium citrate resulted in the highest yield of 349.37 mg/L, representing a 24.1% increase. Although sodium citrate does not directly participate in polyketide chain assembly, as an important intermediate of the TCA cycle, it may indirectly enhance secondary metabolic flux by increasing the intracellular supply of acetyl-CoA and malonyl-CoA, thereby promoting Ppd A biosynthesis. Moderate supplementation with L-tyrosine enhanced both Ppd A production and biomass accumulation. At a concentration of 0.5 g/L, Ppd A production increased by 16.1% compared with the control without L-tyrosine. However, when the concentration exceeded 0.5 g/L, Ppd A production decreased. Therefore, 0.5 g/L was determined to be the optimal concentration for L-tyrosine supplementation.

To further optimize the timing of precursor addition, sodium citrate (2 g/L) and L-tyrosine (0.5 g/L) were added at different fermentation times (Fig. 3E and F). The results showed that supplementation with sodium citrate at 72 h of fermentation resulted in the highest Ppd A yield and maximum dry cell weight. In contrast, the highest Ppd A production was achieved when L-tyrosine was added at 24 h, with an increase of approximately 13.1% compared with the control, accompanied by a slight increase in biomass.

The transcriptional levels of genes within the Ppd A biosynthetic gene cluster were further analyzed. Upon sodium citrate supplementation, *dmfC*, *dmfF*, and *dmfG* were all significantly upregulated (Fig. 3G). Among these genes, *dmfC* encodes an enoyl reductase responsible for the reductive modification of the polyketide chain. Given the potential stimulatory effect of sodium citrate on central carbon metabolism and polyketide precursor supply, the upregulation of *dmfC* implies that greater polyketide precursor availability may create higher demand for reductive modification. *dmfF* is predicted to encode a cluster-localized transcription factor, whereas *dmfG* encodes an α/β hydrolase potentially involved in downstream processing of biosynthetic intermediates. Their simultaneous upregulation indicates that sodium citrate supplementation can influence both the transcriptional regulation of this biosynthetic cluster and pathway-related enzymatic reactions. Following supplementation with L-tyrosine, a structural precursor of Ppd A, *dmfF* and *dmfG* were markedly upregulated (Fig. 3H). This observation suggests that the stimulatory effect of L-tyrosine is mainly linked to structural precursor provision, transcriptional control of the biosynthetic gene cluster, and downstream processing of biosynthetic intermediates. Collectively, both supplements elevated the transcript levels of *dmfF* and *dmfG*, demonstrating that improved Ppd A production is associated with not only precursor supply but also the transcriptional activity of its biosynthetic gene cluster.

#### Addition of metal ions

Metal ions play critical roles in regulating fungal hyphal morphology, enzyme activity, energy metabolism, redox balance, and secondary metabolism (Vasudhevan et al. 2024_)._ Therefore, a series of representative metal ions commonly used in microbial fermentation, including Cu^2+^, Ca^2+^, Mn^2+^, Zn^2+^, Mg^2+^, Fe^2+^, Al^3+^, and Fe^3+^, were selected to systematically evaluate the effects of divalent and trivalent metal ions on Ppd A production and biomass accumulation (Fig. 3I). The results showed that the addition of Zn^2+^ significantly enhanced Ppd A biosynthesis, increasing the production to 329.26 mg/L. Meanwhile, the biomass increased to 14.32 g/L, indicating the addition of Zn^2+^ promoted both product formation and growth. This effect may be attributed to the involvement of Zn^2+^ in RNA polymerase activity, whereby exogenous Zn^2+^ supplementation potentially activates the transcription of secondary metabolite biosynthetic gene clusters (Prasad 2008). In addition, zinc chloride, as a Lewis acid, may favor fungal growth under mildly acidic conditions, thereby indirectly promoting Ppd A accumulation (Zhu et al. 2022). Although Mg^2+^ and Fe^2+^ were identified as potentially important factors in the preliminary screening experiments (Fig. 1J), their exogenous addition under the present experimental conditions resulted in a reduced Ppd A production, which is likely due to the applied concentrations exceeding their optimal ranges and exerting inhibitory effects on metabolic processes. Moreover, the addition of Cu^2+^ and Mn^2+^ markedly suppressed Ppd A production while slightly increasing biomass. Overall, among the tested metal ions, Zn^2+^ was the most effective in enhancing Ppd A production.

The concentration of Zn^2+^ supplementation had a marked effect on Ppd A production (Fig. 3J). As the Zn^2+^ concentration increased from 0.1 to 2 mmol/L, both Ppd A production and fungal dry weight gradually increased. When the concentration was further increased to 3–5 mmol/L, the fungal dry weight remained relatively high, whereas Ppd A production decreased. Therefore, 2 mmol/L was selected as the appropriate supplementation concentration. Screening of the supplementation time showed that Ppd A production was relatively low when Zn^2+^ was added at 0–24 h, whereas supplementation at 48–72 h significantly promoted Ppd A accumulation, with the highest production observed at 72 h (Fig. 3K). Further delaying Zn^2+^ supplementation resulted in a gradual decrease in Ppd A production, which declined to 215.12 mg/L when Zn^2+^ was added at 144 h. RT-qPCR analysis showed that, under the optimal supplementation conditions, *dmfF* and *dmfG* were significantly upregulated (Fig. 3L), suggesting that Zn^2+^ may promote the activation of Ppd A biosynthesis and the hydrolytic release of the product. The transcriptional level of *dmfD* increased to 1.84-fold that of the control. *dmfD* encodes a major facilitator superfamily (MFS) transporter, which is generally involved in the transmembrane transport of small molecules or metabolites, suggesting that Zn^2+^ supplementation may enhance substrate transport across the cell membrane.

#### Addition of surfactants

Surfactants can enhance membrane permeability and promote the dispersion of solid particles, thereby effectively improving mass transfer and oxygen transfer within the fermentation system (Lee et al. 2017). Four surfactants (sorbitol, DMSO, Tween 80, and n-dodecane) were tested for their effects on Ppd A production (Fig. 3M). DMSO and sorbitol significantly increased yield, with DMSO enhancing Ppd A by nearly 32% and slightly increasing biomass. n-dodecane, acting as a hydrophobic oxygen carrier, may improve the dissolved oxygen conditions for product formation by enhancing oxygen transfer efficiency between the organic and aqueous phases. However, it did not significantly change Ppd A yield. The addition of Tween 80 markedly decreased Ppd A to 68.46 mg/L with no noticeable biomass change. Overall, DMSO was identified as the most effective additive under current conditions.

Further optimization of DMSO concentration and addition timing revealed trends in Ppd A production and biomass (Fig. 3N and O). As DMSO concentration increased, Ppd A production initially increased and then decreased, while biomass first decreased then increased, reaching maximum Ppd A production at 1 g/L with minimum biomass. Addition at 72 h resulted in the highest Ppd A production, increasing 24.4% over control group, with biomass at 15.60 g/L. The results indicate a balance between product formation and cell growth. Therefore, adding 1 g/L DMSO at 72 h efficiently enhanced Ppd A production.

#### Combined addition

Four exogenous compounds (ZnCl₂, L-tyrosine, DMSO, and sodium citrate) were found to effectively improve Ppd A production. Sixteen different combination trials at varying concentrations were conducted (Fig. 3P). The combined addition of DMSO and sodium citrate exhibited the most effect, achieving a Ppd A yield of 364.99 mg/L, which is 5.68-fold higher than that in M3 medium.

### Effect of mycelial morphology on Ppd A synthesis

#### Effect of nitrogen source on morphology

**Fig. 4:**
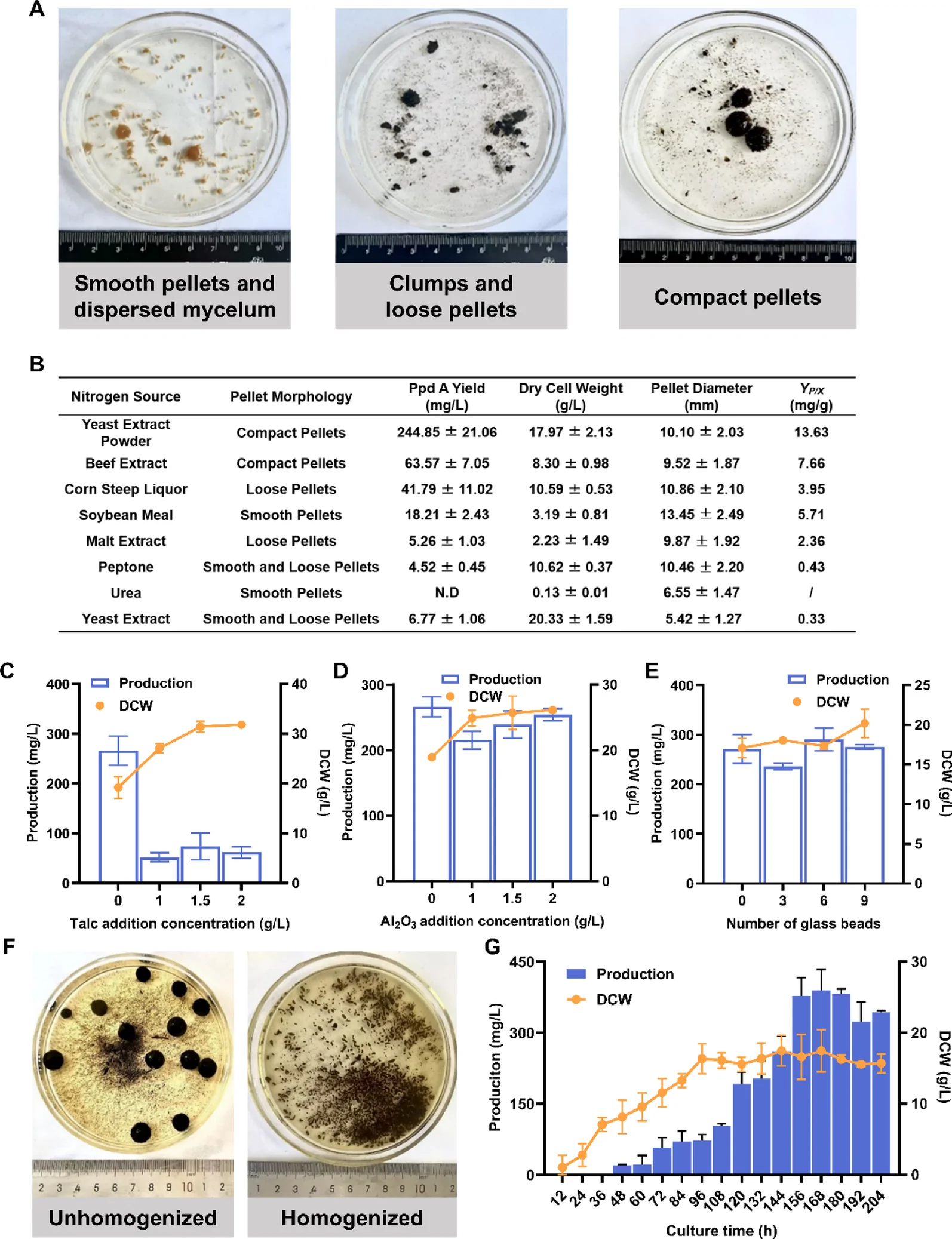
Effects of nitrogen sources and shear stress on mycelial morphology of *Didymella* sp. FATR0054 and Ppd A production. **A** Schematic illustration of representative mycelial morphologies of *Didymella* sp. FATR0054 under different nitrogen sources. **B** Effects of different nitrogen sources on mycelial morphology, pellet diameter, Ppd A production, and dry cell weight of *Didymella* sp. FATR0054. Effects of talc (**C**), Al_2_O_3_ (**D**) and glass beads (**E**) on Ppd A production in shake-flask cultures. **F** Morphological changes of the pellets during fermentation inoculated with unhomogenized or homogenized seed cultures. **G** Growth and Ppd A production profiles of *Didymella* sp. FATR0054 after inoculation with pre-homogenized seed culture

Fungal morphology is a key determinant of secondary metabolite production. Nitrogen sources not only significantly influence growth performance but also directly affect morphology and product synthesis (Park et al. 1999; Wang et al. 2021). Figure 4A shows three typical morphologies of the fungus *Didymella* sp. FATR0054. When cultivated with different nitrogen sources, the fungus formed smooth pellets with dispersed hyphae, clumps with loose pellets, and compact pellets, respectively.

As shown in Fig. 4B, when yeast extract powder and beef extract were used as nitrogen sources, the fungus formed compact pellets. Among these, the yeast extract powder group exhibited the highest degree of pellet compactness, corresponding to relatively high levels of both Ppd A production and dry cell weight. In contrast, when corn steep liquor and malt extract were used as nitrogen sources, the fungus formed loose pellets, and the Ppd A yield decreased significantly. When peptone and yeast extract were used as nitrogen sources, the fungus exhibited a smooth and loose pellet morphology. Although the pellet diameter was large (10–20 mm), the product accumulation capacity was relatively low.

These results indicate that the type of nitrogen source in the medium simultaneously affected pellet morphology, biomass, and Ppd A production. The yeast extract powder group formed compact pellets and achieved the highest Ppd A production. However, the beef extract group, which also formed compact pellets, exhibited relatively low production. Therefore, pellet compactness was associated with high Ppd A production but was not the sole determinant of Ppd A yield. The nutritional composition of the nitrogen source, together with pellet size, compactness, and uniformity, may collectively determine Ppd A accumulation.

#### Effect of mechanical shear on growth and production

In filamentous fungal fermentation, inert particle supplementation can modulate mycelial aggregation and the associated shear environment. These particles play an important role in regulating fungal morphology by providing shear force to inhibit excessive pellet growth and improve pellet dispersion, thereby enhancing the mass transfer efficiency of oxygen and nutrients (Yang et al. 2016). Therefore, three types of particulate materials (talc, Al_2_O_3_, and glass beads) were added to the culture system to modify mycelial morphology (Fig. 4C–E). It was found that the addition of the three types of particles promoted biomass accumulation, but did not improve the product yield. In particular, the addition of talc led to a significant reduction in yield.

To further optimize mycelial morphology, the seed culture was subjected to a pre-grinding treatment. The morphological characteristics of the pellets formed before and after treatment are shown in Fig. 4F. After homogenization, the pellet diameter decreased from 10 mm to approximately 1 mm. The pellets became uniform in morphology, and pellet compactness increased significantly. As shown in Fig. 4G, under the combined conditions of the high-yield medium and the pre-grinding treatment of the seed culture, the yield of Ppd A reached 389.25 mg/L, representing a 34.52% increase compared with the group without seed grinding shown in Fig. 2G.

### CFD-based elucidation of shear effects on morphology and Ppd A synthesis

**Fig. 5:**
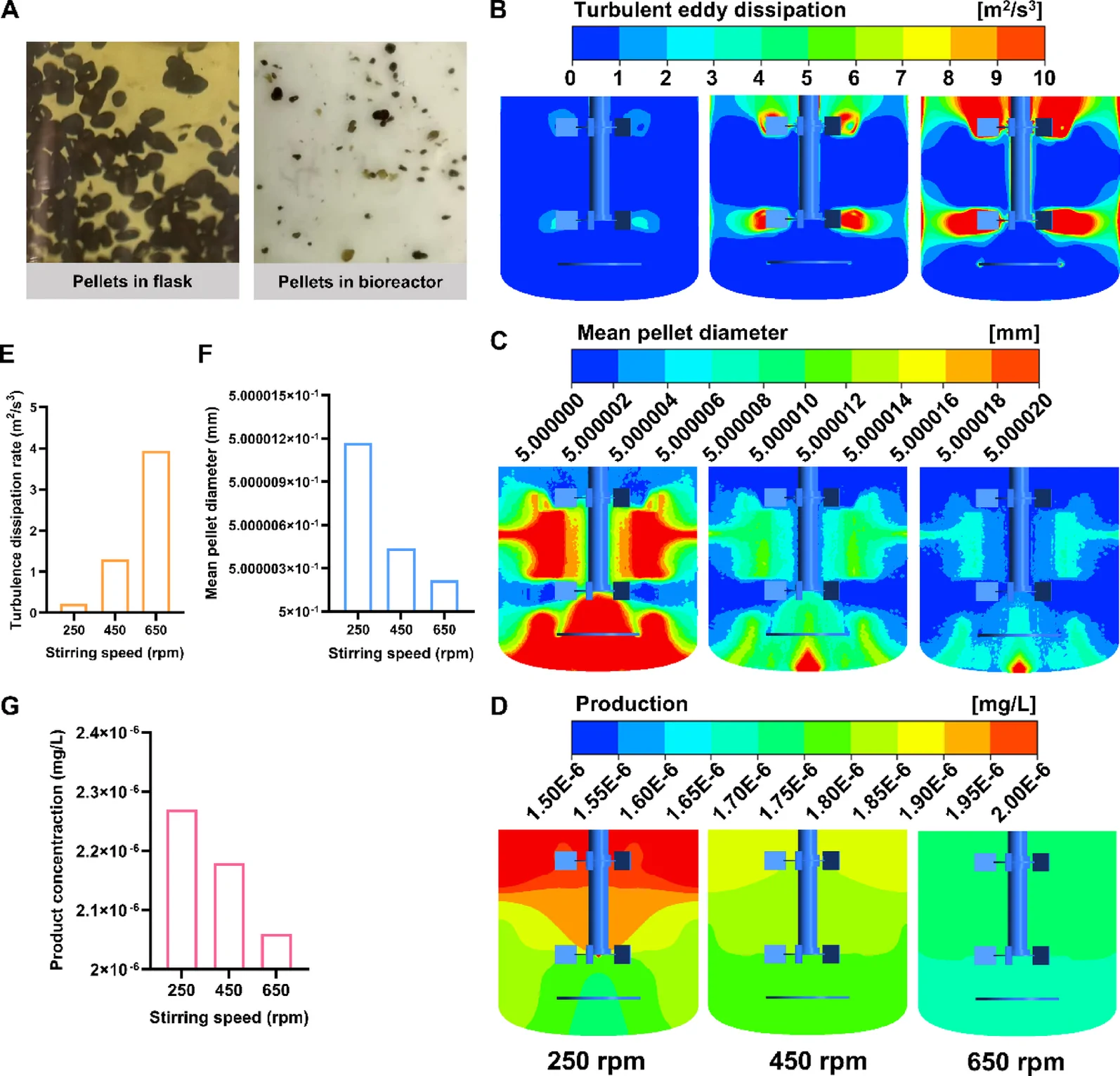
CFD-based analysis of hydrodynamic conditions, mycelial morphology, and Ppd A production in a 5-L bioreactor at different agitation speeds. **A** Comparison of mycelial morphology of *Didymella* sp. FATR0054 in shake-flask cultures and a 5-L bioreactor. Contour distributions of turbulent dissipation rate (**B**), mean pellet diameter (**C**), and Ppd A production (**D**) in the 5-L bioreactor operated at different agitation speeds. Volume-averaged values of turbulent energy dissipation rate (**E**), mean pellet diameter (**F**), and Ppd A production (**G**) in the 5-L bioreactor at different agitation speeds

Results from “Effect of nitrogen source on morphology” Section indicated that compact pellets are suitable for product synthesis and mycelial growth. However, pellet size was not completely uniform throughout the fermentation process. In shake flasks, the diameter of fungal pellets is relatively uniform. However, in a bioreactor, due to the effects of shear force and substrate gradients, the pellet diameter is significantly more heterogeneous and is smaller than that in shake flasks (Fig. 5A). To further reveal the mechanism by which shear in a 5-L bioreactor influences the morphological evolution of the fungal pellets and the synthesis of Ppd A, a kinetic model for fungal pellet growth was constructed (see Supplementary Material for details) and CFD simulation was used to analyze the flow field in the 5-L bioreactor.

The simulation results showed that as the agitation speed increased, the turbulent dissipation energy in the bioreactor increased significantly, mainly concentrated around the impellers and in the space between the impellers and the baffles (Fig. 5B). As shown in Fig. 5C, at 250 rpm, the overall flow field remained relatively stable, with intense turbulence confined to limited local regions. Under this condition, fungal pellets grew rapidly and developed relatively large diameters; however, excessively large pellets may suffer from oxygen and substrate mass-transfer limitations within the pellet interior. At 450 rpm, the turbulent energy dissipation rate increased in the vicinity of the impellers. The initially larger pellets located between the two impeller layers and near the vessel bottom were reduced in size due to enhanced shear, resulting in a more uniform pellet size distribution throughout the bioreactor. When the agitation speed was further increased to 650 rpm, both turbulence intensity and shear stress increased additionally, leading to extensive pellet fragmentation near the impellers and agitation shaft and, consequently, reduced pellet size uniformity. In contrast, the spatial distribution of Ppd A became progressively more uniform with increasing agitation speed (Fig. 5D), an effect attributable to the higher power input and shorter mixing time, allowing the concentration field within the reactor to approach uniformity more rapidly.

According to the kinetic analysis of pellet growth and product formation, pellet growth rate is an important factor affecting the Ppd A formation rate. Faster pellet growth leads to higher biomass accumulation and, accordingly, a higher rate of Ppd A formation. The volume-averaged turbulent energy dissipation rate, pellet diameter, and product concentration after 20 s of simulation showed that, with increasing agitation speed, turbulent energy dissipation increased continuously, whereas the mean pellet diameter and Ppd A production decreased (Fig. 5E–G). Thus, although a higher agitation speed can improve the uniformity of the concentration field within the bioreactor, excessive agitation may be unfavorable for biomass growth and maintaining a uniform pellet size distribution. Notably, the reduction in Ppd A production from 450 to 650 rpm was greater than that observed from 250 to 450 rpm. Taken together, considering the concentration field, pellet size distribution, and mass-transfer performance, 450 rpm provided a more favorable balance between hydrodynamic shear and its effects on fungal pellet morphology.

Accordingly, during bioreactor cultivation of the fungus, the agitation speed during the exponential growth phase should be gradually increased in coordination with dissolved oxygen levels. During the stationary phase, 450 rpm may serve as a candidate operating condition that balances mixing performance, local shear, and energy consumption. Further increases in agitation speed may intensify pellet fragmentation in the impeller region and increase pellet size heterogeneity. In the later stage of fermentation, oxygen transfer may instead be maintained by increasing the aeration rate. Under such operating conditions, a favorable balance can be achieved among turbulent energy dissipation, shear intensity, fungal pellet morphology, and Ppd A production.

## Discussion

In our previous work, structurally unique hirsutellone-type macrocyclic alkaloid Phomapyrrolidone A, exhibiting significant anti-triple-negative breast cancer (TNBC) activity both in vitro and in vivo, along with favorable biosafety, was isolated from the fermentation products of the marine-derived fungus *Didymella* sp. FATR0054. It has potential as a lead molecule for novel anti-TNBC drugs. To enhance the biosynthetic capacity of the strain and establish a production basis for subsequent pharmacological evaluation and drug development, fermentation optimization was carried out from four perspectives: development of a high-yield medium, exogenous supplementation, morphological regulation, and optimization of the hydrodynamic environment. During this process, biomass accumulation and Ppd A production were found not to exhibit a simple positive correlation under different cultivation conditions. Certain nitrogen sources or inert particles promoted fungal growth but reduced Ppd A production. The growth profile further showed that Ppd A continued to accumulate during both the exponential and stationary phases, indicating a partially growth-associated product formation pattern. Thus, Ppd A production appears to depend on the coordinated effects of precursor availability, activation of the biosynthetic gene cluster, and oxygen and substrate transfer within and around fungal pellets.

In the exogenous supplementation experiments, sodium citrate, L-tyrosine, and Zn^2+^ all enhanced Ppd A production and induced distinct transcriptional responses within the *dmf* biosynthetic gene cluster. Sodium citrate increased the transcriptional levels of *dmfC*, *dmfF*, and *dmfG*, whereas L-tyrosine mainly upregulated *dmfF* and *dmfG*, and Zn^2+^ predominantly increased the expression of *dmfF*, *dmfG*, and *dmfD*. *dmfF* encodes a cluster-associated transcriptional regulator, whereas *dmfG* encodes an α/β hydrolase. The consistent upregulation of these two genes in response to all three supplements suggests that they may represent important regulatory nodes involved in coordinating Ppd A biosynthesis. DmfF functions as a positive transcriptional regulator, and increased expression of this regulator may activate transcription of the entire biosynthetic gene cluster and thereby enhance secondary metabolite production (Bergman et al. 2010). The DmfG homolog PydG has been reported to be essential for intramolecular Knoevenagel condensation of the reductively released intermediate and subsequent formation of the 3-pyrrolin-2-one ring (Ohashi et al. 2021). Accordingly, upregulation of *dmfG* may facilitate formation of the nitrogen-containing heterocyclic scaffold characteristic of hirsutellone-type compounds. *dmfC* encodes a trans-acting enoyl reductase involved in reductive modification of the polyketide chain. Sodium citrate may increase the intracellular supply of acetyl-CoA and its downstream precursor malonyl-CoA while simultaneously enhancing *dmfC* expression, suggesting that improved precursor supply from central carbon metabolism may be accompanied by increased capacity for reductive modification of the polyketide chain. Following Zn^2+^ supplementation, the upregulation of *dmfD* suggests that the encoded major facilitator superfamily (MFS) transporter may participate in the transmembrane transport of precursors or products, although its specific substrate and transport direction remain to be determined. Overall, the increase in Ppd A production was consistent with the elevated transcriptional levels of key genes within the biosynthetic gene cluster, indicating that the stimulatory effects of exogenous supplements may involve not only precursor supplementation but also activation of the biosynthetic gene cluster and redistribution of metabolic flux. These findings provide candidate targets for subsequent metabolic engineering. Future efforts could focus on enhancing *dmfF* expression, co-expressing *dmfC* and *dmfG*, and elucidating the transport function of *dmfD* to construct high-producing engineered strains.

In addition to metabolic precursor supply and transcriptional regulation of the biosynthetic gene cluster, the type of nitrogen source exerts a significant influence on secondary metabolite biosynthesis in filamentous fungi. Different nitrogen sources not only affect mycelial growth and intracellular nutritional status but can also modulate oxygen and nutrient transfer efficiency by altering hyphal growth patterns and mycelial morphology (Zheng et al. 2025). Comparison with previous studies suggests that the effects of nitrogen sources on mycelial morphology, including branching patterns and pellet compactness, are not universal but may vary among fungal genera or species (Wang et al. 2021). Accordingly, the morphological characteristics favorable for product biosynthesis may also differ among fungal systems. In this study, organic nitrogen sources, such as yeast extract powder, favored the formation of compact pellets and promoted Ppd A production. In contrast, plant-derived nitrogen sources (e.g., corn steep liquor and malt extract) led to loose pellets and insufficient product. These differences may be related to variations in the amino acids, peptides, vitamins, trace elements, and effective C/N ratios provided by different nitrogen sources, which may in turn influence cell wall biosynthesis and hyphal surface interactions. The pellet architecture of filamentous fungi is primarily determined by hyphal arrangement patterns and cell wall composition, in which chitin and α/β-1,3-glucan serve as key components for maintaining structural stability of the hyphae (Yoshimi et al. 2017). Previous studies have shown that, compared with dispersed mycelial forms, compact pellets possess higher contents of cell wall polysaccharide components, thereby exhibiting greater mechanical stability and shear resistance (Gromozova et al. 2025). Furthermore, modulation of gene expression related to cell wall biosynthesis can alter pellet size and compactness, subsequently influencing secondary metabolite formation (Ost et al. 2025). Accordingly, we hypothesize that different nitrogen sources may affect hyphal aggregation patterns and pellet structural characteristics by modulating the nutritional status of the organism and the cell wall formation process. Compact pellets may reduce the entanglement of freely dispersed hyphae, alleviate the non-Newtonian flow behavior of the fermentation broth, and provide a relatively stable microenvironment for metabolically active hyphae at the pellet periphery. Therefore, compared with excessively loose and fragile pellets, compact pellets may be more favorable for sustaining Ppd A biosynthesis. However, the present results also indicate that pellet compactness alone is not sufficient to ensure high production. Beef extract likewise promoted the formation of compact pellets, yet Ppd A production was much lower than that obtained with yeast extract. Another yeast-derived nitrogen source supported relatively high biomass accumulation but only limited Ppd A production. These observations suggest that Ppd A production is jointly determined by the metabolic composition provided by the nitrogen source and an appropriate fungal morphology. Excessively large compact pellets can increase the diffusion distance for oxygen and substrates into the pellet interior, potentially resulting in oxygen limitation, nutrient deprivation, and even loss of viability in the central region. In this study, the initial pellet diameter was approximately 10 mm. Homogenization of the seed culture before inoculation reduced the pellet diameter to approximately 1 mm and improved size uniformity, ultimately increasing Ppd A production by 34.52%. The reduction in pellet size markedly increased the total pellet surface area, suggesting improved mass transfer between the pellet interior and the surrounding fermentation environment. Therefore, pellets with an appropriate degree of compactness, controlled size, and a relatively uniform size distribution may provide a more favorable morphology for Ppd A biosynthesis by balancing structural stability with efficient mass transfer.

In addition, no obvious changes in pellet size or morphology were observed following supplementation with metabolic precursors, metal ions, or surfactants. Their stimulatory effects on Ppd A production are therefore more likely to arise from changes in membrane properties, intracellular metabolism, or gene transcription rather than from alterations in pellet morphology. Costes et al. (2021) reported that DMSO can alter the expression of secondary metabolite biosynthetic gene clusters without significantly affecting fungal growth or macroscopic morphology. In contrast, Tween 80 has been reported to promote the formation of smaller mycelial pellets in some fungi (Bellaouchi et al. 2024). The variation in morphological responses among studies may reflect differences in fungal species and culture conditions.

Based on the CFD-PBM simulation results, 450 rpm can be regarded as a reasonable operating compromise for the current system when pellet size distribution, product concentration distribution, local energy dissipation, and reactor energy consumption are considered collectively. At 250 rpm, the lower local shear and fragmentation intensity favored biomass growth but also promoted the formation of larger pellets. In addition, according to the general relationship between agitation intensity and the volumetric oxygen transfer coefficient, both mixing performance and kLa are typically lower at 250 rpm than at 450 rpm. Increasing the agitation speed to 650 rpm could further reduce pellet size and enhance external mass transfer, but at the expense of higher local energy dissipation, a greater risk of excessive pellet fragmentation in the impeller region, and increased energy consumption. Pellet fragmentation and morphological changes in filamentous fungi are highly sensitive to the local shear environment. Therefore, from the perspective of balancing mixing, mass transfer, morphological uniformity, and shear-induced fragmentation, 450 rpm represents the most reasonable agitation condition for the present system, although the CFD model did not predict the highest Ppd A formation at this speed. In fact, the CFD model incorporating pellet growth and product formation predicted the instantaneous product formation rate in the order of 250 rpm > 450 rpm > 650 rpm. This trend mainly arose because the product source term in the model was coupled to the fungal growth rate, and the highest biomass formation rate was predicted at 250 rpm. However, the current model does not account for intra-pellet diffusion of oxygen and substrates, pellet porosity, or the effective metabolically active volume within the pellets. Consequently, larger pellets are associated with greater biomass in the model, which may lead to overestimation of product formation at 250 rpm. Moreover, in actual bioreactor cultivation, lower agitation speeds generally result in a lower gas–liquid mass-transfer coefficient (Zhou et al. 2027), thereby limiting oxygen availability to the fungus. Future models should therefore incorporate reaction–diffusion equations for oxygen and substrates within fungal pellets to improve the accuracy of product formation predictions. Another limitation of the present CFD model is that pellet growth, fragmentation, and product formation were simulated using a transient approach. Because transient simulations are computationally expensive, only second-scale changes in pellet growth and product formation could be simulated in the present study. Relative to a fungal fermentation process lasting several days, this time window is extremely short, and consequently only minor changes in mean pellet size and Ppd A concentration were observed under the different agitation conditions. Although these simulations cannot be interpreted as predictions of final pellet size or product titer over the full fermentation cycle, the differences in instantaneous formation rates can still be used to compare the relative trends among agitation conditions under identical initial conditions. Future work should further optimize the numerical strategy and time-step settings to achieve a better balance between predictive accuracy and computational efficiency.

The pellet growth-fragmentation and product formation model established in this study is based on governing relationships among local turbulent energy dissipation, pellet growth and breakage, and product formation, and is therefore not inherently restricted to the 5-L bioreactor scale. Previous studies have combined CFD with fungal growth kinetics or population balance models for scale-up analysis of filamentous fungal bioreactors (Cappello et al. 2021). However, during scale-up, it is difficult to simultaneously maintain mixing time, gas holdup, kLa, power input per unit volume, impeller tip speed, and local maximum energy dissipation, while pellet breakage parameters may also vary with impeller configuration and reactor scale. Future studies could develop hydrodynamic models for pilot-scale or larger-scale bioreactors, using the kLa, power input per unit volume, and the distribution of local energy dissipation rates as key scale-up constraints. Coupling these models with pellet size distributions and Ppd A production kinetics could enable the prediction of fermentation performance and guide stepwise bioreactor scale-up.

## Conclusion

This study focused on the marine-derived fungus *Didymella* sp. FATR0054 and established an efficient fermentation optimization system for its secondary metabolite, Ppd A. By analyzing the nutritional requirements of the strain and the relationship between morphology and metabolism, combined with hydrodynamic analysis, a systematic improvement from medium optimization to bioreactor scale-up was achieved.

First, systematic optimization of medium composition and fermentation parameters increased the shake-flask yield of Ppd A from 4.52 mg/L to 289.36 mg/L, and the optimized process was successfully scaled up from shake flasks to a bioreactor. With further exogenous addition of DMSO and sodium citrate to regulate transcriptional processes and product release, the yield was increased to 364.99 mg/L.

Furthermore, the study revealed that regulating mycelial morphology was a key factor in increasing yield. Pre-grinding the seed culture controlled the pellet diameter from approximately 10 mm to about 1 mm, resulting in compact and uniform pellets. Under this morphology, the pellets avoided both internal mass transfer limitations and cell autolysis caused by excessive size, as well as the loss of metabolic activity caused by excessive fragmentation. Ppd A production further increased to 389.25 mg/L, representing an 86-fold improvement over the initial yield and the highest fermentation level reported to date. CFD simulations indicated that in the 5-L bioreactor, an agitation speed of 450 rpm provided a moderate shear environment. This improved mass transfer while maintaining structural stability of the mycelium, achieving an optimal balance between production and morphological stability.

In summary, this study established a systematic optimization strategy for Ppd A production. By combining experimental validation and CFD simulation, it provides a new theoretical basis for morphological regulation and fermentation process modeling in filamentous fungi. It also offers an important reference and guidance for the efficient fermentation and process scale-up of secondary metabolites from other marine-derived fungi.

## Supplementary Information

Below is the link to the electronic supplementary material.

Supplementary Material 1

## Data Availability

The data that support the findings of this study are available from the corresponding author upon reasonable request.
