# Supplementary material for: Enhanced production of phomapyrrolidone a through culture-condition optimization and morphological regulation in Didymella sp. FATR0054
Source: Bioresour Bioprocess. 2026 Sep 21;13(1):139. doi: 10.1186/s40643-026-01132-2 (PMC13590468; doi:10.1186/s40643-026-01132-2)
Supplement: Supplementary file 1 [file 40643_2026_1132_MOESM1_ESM.docx]

**Supplementary Materials**

**Enhanced Production of Phomapyrrolidone A through Culture-Condition Optimization and Morphological Regulation in *Didymella* sp. FATR0054**

Weiyan Zhou ^1^, Zhongyuan Chen ^1^, Jiacheng Guo ^1^, Yiming Yang ^1^, Fan Yang ^3，4^， Xiujuan Xin ^1^, Liwei Zhuang ^2,*^, Lizhi Gong ^1,*^, Faliang An ^1, 3,*^

^1^ State Key Laboratory of Bioreactor Engineering, East China University of Science and Technology, 130 Mei Long Road, Shanghai 200237, China

^2^ School of Chemical Engineering, East China University of Science and Technology, Shanghai 200237, China

^3^ Marine Biomedical Science and Technology Innovation Platform of Lin-Gang Special Area, No. 4, Lane 218, Haiji Sixth Road, Shanghai 201306, China

^4^ Department of Pharmacy, Renji Hospital, School of Medicine, Shanghai Jiao Tong University, Shanghai 200127, China; bill1985@126.com

^*^ Corresponding author: Liwei Zhuang, Lizhi Gong, Faliang An

*E-mail* address: [lwzhuang@ecust.edu.cn](mailto:lwzhuang@ecust.edu.cn) (L. Z.), [15216603669@163.com](mailto:15216603669@163.com)(L. G.), [flan2016@ecust.edu.cn](mailto:flan2016@ecust.edu.cn) (F. A.)

**1. Parameter estimation of the kinetic model**
1.1 Growth rate of pellet diameter

The pellet diameters were divided into six intervals: 0-1 mm, 1-2 mm, 2-3 mm, 3-4 mm, 4-5 mm, and 5-6 mm. During 250 mL shake-flask fermentation, the seed culture was homogenized by grinding and inoculated at 5% (v/v) into the optimized fermentation medium described in Section 2.1.2. Cultivation was carried out at 28 ℃ and 200 r/min, and the number of pellets within each size interval was recorded. The average pellet diameter at each fermentation time point was calculated using Eq. (1), and an exponential function was fitted to the relationship between the average diameter and fermentation time, thereby yielding the growth rate equation of pellet diameter (Eq. (2)).

$$\text{D}_{\text{ave}}\text{=}\frac{{\sum\text{D}}_{\text{mid}}\text{*}\text{N}}{\text{N}}$$

(S1)

$$\text{D}\text{=0.23388}\text{e}^{\frac{\text{t}}{\text{105.30683}}}\text{+0.20695}$$

(S2)

**Fig.S1 Fitting of growth rate of the diameter of pellets**

It should be noted that this growth-rate correlation was fitted from shake-flask data, in which pellet size is relatively uniform; in the stirred-tank bioreactor the pellet diameter is more heterogeneous owing to shear and substrate gradients, so the fitted model is used here as an empirical growth-rate input for the CFD-PBM simulation rather than as a scale-independent description of pellet size.

1.2 Kinetic model of biomass growth

The biomass growth model describes the self-growth behavior of *Didymella* sp. FATR0054 by correlating changes in cell dry weight during fermentation. This model effectively captures the growth characteristics of filamentous microorganisms throughout the fermentation process.

$$\mu=\frac{dX}{dt}=\mu_{m}(1-\frac{X}{X_{m}})X$$

(S3)

where the variable *X* represents the biomass concentration, *X*_m_ denotes the maximum biomass concentration, and *μ* represents the specific growth rate of the biomass, which is assumed to be constant (*μ* is related to the microbial strain, cultivation temperature, and other limiting substrates). Under the initial condition *t* = 0, *X* = *X_0_*, the integrated form of this equation is given as:

$$X\left( t \right)=\frac{X_{0}e^{\mu t}}{1-\frac{X_{0}}{X_{m}}(1-e^{\mu t})}$$

(S4)

1.3 Kinetic model for Ppd A production by *Didymella* sp. FATR0054

Based on the relationship between Ppd A production and the growth characteristics of *Didymella* sp. FATR0054, a classical product formation kinetic model was selected, and the corresponding equation is expressed as follows:

$$\frac{dP}{dt}=\beta X+\gamma\frac{dX}{dt}$$

(S5)

where *P* represents the Ppd A titer, *γ* denotes the product formation rate associated with the biomass growth rate, and *β* represents the product formation rate independent of the biomass growth rate.

1.4 Pellet breakage model

$$\text{ }\text{S}\text{=}\text{a}\left( \frac{\text{ε}}{\text{b}} \right)^{\text{c}}\text{L}^{\text{d}}$$

(S6)

where *S* denotes the pellet breakage rate, *ε* represents the turbulent energy dissipation rate, and *L* is the pellet diameter. The determination of the coefficients *a*, *b*, *c*, and *d* was based on the results reported by Lehr *et al*^[1]^ and Li *et al*^[2]^*.*

**Table S1. Fitted values of kinetic parameters**

| parameters | unit | value |
| --- | --- | --- |
| *μ* | h^-1^ | 0.05755 |
| *X_0_* | g/L | 0.78 |
| *X_m_* | g/L | 17.46 |
| *β* | - | 0.2784 |
| *γ* | - | 6.625 |
| *a* | - | 0.001 |
| *b* | - | 0.001 |
| *c* | - | 0.6 |
| *d* | - | -1.5 |

**Table S2. Medium formula**

| Medium | Medium Composition |
| --- | --- |
| M1 | MgSO₄ 0.5 g/L, peptone 10 g/L, yeast extract 2 g/L, KH₂PO₄ 1 g/L, glucose 30 g/L. |
| M2 | Soybean meal 15 g/L, soluble starch 20 g/L, peptone 2 g/L, yeast extract 2 g/L, artificial sea salt 30 g/L. |
| M3 | FeSO₄·7H₂O 0.01 g/L, K₂HPO₄ 1 g/L, glucose 20 g/L, yeast extract 1 g/L, NaNO₃ 3 g/L, KCl 1 g/L, MgSO₄·7H₂O 0.5 g/L. |
| M4 | Glucose 20 g/L, malt extract 20 g/L, peptone 1 g/L, MgSO₄ 0.5 g/L, KH₂PO₄ 0.5 g/L, sea salt 2 g/L. |
| M5 | Sucrose 20 g/L, yeast extract 10 g/L, artificial sea salt 10 g/L. |
| M6 | Maltodextrin 20 g/L, polypeptone 10 g/L, yeast extract 5 g/L, MgSO₄ 0.5 g/L, KH₂PO₄ 5 g/L. |
| M7 | Soluble starch 50 g/L, yeast extract 10 g/L, KH₂PO₄ 1 g/L, FeSO₄·7H₂O 0.01 g/L, MgSO₄ 0.5 g/L, sea salt 30 g/L. |
| M8 | Malt extract 20 g/L, peptone 3 g/L, artificial sea salt 15 g/L. |
| M9 | Glucose 20 g/L, malt extract 3 g/L, peptone 5 g/L, yeast extract 3 g/L, NaCl 10 g/L. |
| M10 | Soybean meal 15 g/L, glucose 30 g/L, peptone 2 g/L, yeast extract 2 g/L, NaCl 10 g/L. |
| M11 | Soluble starch 120 g/L, KH₂PO₄ 1 g/L, corn flour 2.5 g/L, (NH_4_)_2_SO_4_ 5 g/L, CaCO_3_ powder 20 g/L, NaCl 5 g/L, NaNO₃ 10 g/L. |

Note: Media containing glucose were sterilized at 115 °C for 30 min; media without glucose were sterilized at 121 °C for 30 min.

**Table S3. List of reagents**

| Name | Supplier |
| --- | --- |
| Peptone | Shanghai Sangon Biotech |
| Agar Powder (AR) | Shanghai Sangon Biotech |
| Beef Extract (BR) | Shanghai Sangon Biotech |
| Sodium Chloride (AR) | Shanghai Titan Scientific |
| Malt Extract | Shanghai Titan Scientific |
| Soluble Starch (AR) | Shanghai Titan Scientific |
| Methanol (AR) | Shanghai Titan Scientific |
| Sodium Nitrate (AR) | Shanghai Titan Scientific |
| Sodium Citrate | Shanghai Titan Scientific |
| Sodium Acetate | Sinopharm Chemical Reagent |
| Sodium Malonate | Sinopharm Chemical Reagent |
| Yeast Extract (BR) | Sinopharm Chemical Reagent |
| Magnesium Sulfate Heptahydrate (AR) | Sinopharm Chemical Reagent |
| Potassium Nitrate (AR) | Sinopharm Chemical Reagent |
| Dipotassium Hydrogen Phosphate (AR) | Sinopharm Chemical Reagent |
| Ferrous Sulfate Heptahydrate (AR) | Sinopharm Chemical Reagent |
| Glucose (AR) | Sinopharm Chemical Reagent |
| Potassium Dihydrogen Phosphate (AR) | Sinopharm Chemical Reagent |
| Ammonium Nitrate (AR) | Sinopharm Chemical Reagent |
| Mannitol (AR) | Sinopharm Chemical Reagent |
| Maltose (AR) | Sinopharm Chemical Reagent |
| Sucrose (AR) | Sinopharm Chemical Reagent |
| Hydrochloric Acid (AR) | Sinopharm Chemical Reagent |
| Sodium Hydroxide (AR) | Sinopharm Chemical Reagent |
| Glycerol (AR) | Sinopharm Chemical Reagent |
| Urea | Sinopharm Chemical Reagent |
| Corn Steep Liquor | Wudi Bohao Biotechnology |
| Sea Salt | Weifang Nuanzhong Trading |
| Anhydrous Magnesium Sulfate | Shanghai Lingfeng Chemical Reagent |
| Soybean Powder | Beijing Hongrun Baoshun Technology |
| Cane Molasses | Jinclon (Beijing) Biotechnology |
| Vegetable Oil | Yihai Kerry Arawana Holdings |
| Yeast Extract Powder | Angel Yeast |
| Name | Supplier |
| NCM Biotech RNA kit M5105 | NCM Biotech |
| Hifair® III 1st Strand cDNA Synthesis SuperMix for qPCR(gDNA digester plus) | Yeasen Biotechnology (Shanghai) Co., Ltd. |
| Hieff UNICON® Advanced qPCR SYBR Master Mix | Yeasen Biotechnology (Shanghai) Co., Ltd. |

**Table S4. Major laboratory instruments**

| Name | Manufacturer |
| --- | --- |
| Laminar Flow Cabinet (SW-CJ-1F) | Suzhou Sujie Purification Equipment |
| Agilent 1260 High Performance Liquid Chromatograph (HPLC) | Agilent Technologies |
| Constant Temperature Drying Oven | Shanghai Experimental Instrument General Factory |
| Floor-standing Incubator Shaker (ZQLY-180) | Shanghai Zhichu Biotechnology |
| Blue Pard Fungal Incubator | Shanghai Yiheng Technology |
| ALP CL-40M Autoclave | ALP Co., Ltd., Japan |
| EYELA Rotary Evaporator | Tokyo Rikakikai Co., Ltd. (EYELA) |
| Constant Temperature Water Bath | Shanghai Yiheng Technology |
| 5-L Bioreactor | Shanghai Guoqiang Biochemical Engineering Equipment |
| Nanodrop 2000 | Thermo Fisher Scientific Inc. |
| CFX96 Touch Real-Time PCR Detection System | Bio-Rad Laboratories, Inc. |

**Table S5. Primer sequences for RT-qPCR**

| Target gene | Primer | Sequence (5’-3’) |
| --- | --- | --- |
| *GAPDH* | Forward  Reverse | GACAAGACCTCGCGCATTTC  CTCGTCAGAGGCGTAAGGAC |
| *dmfA* | Forward  Reverse | CGAGGTCAACGGAACTGTCA  TGTCAGGGACGAGTTTTCCG |
| *dmfB* | Forward  Reverse | GTGAAGTGGATGGGCTCCAA CGTTCCCCAGAAATCCGACA |
| *dmfC* | Forward  Reverse | GCCTTGGTGATACAGTGGCT  AAAAGTGCTCAGGCAAACGC |
| *dmfD* | Forward  Reverse | GCGCCAAAGTTCCAGAAAGG  TCCTAAGTCAGCAACCAGCG |
| *dmfE* | Forward  Reverse | GCGTTGGTATTCACGACTGC  GTGCGAGGCTTTGATTCGTC |
| *dmfF* | Forward  Reverse | TTGTCGTTACCATCCGACCC  TTGTTCTCGCTGTGGGAGTC |
| *dmfG* | Forward  Reverse | TACTCTTCGGCAGCAACTGG  GCTTCTCTGCGCCAATAACG |
| *dmfH* | Forward  Reverse | CGTCAGCATCACTACGCTCA  AAGGTCTCTCGTCCGTCCAA |
| *dmfX* | Forward  Reverse | ACGGCTCTACGCAACATAGC  GGCAAGTCGGTAGTCAGAGC |
| *dmfZ* | Forward  Reverse | ACCGATTTACACCCGCCTTC  CACCCAAAGCAGGACAGAGA |

**Table S6. Reaction system for RT-qPCR**

| Reagents | Volume (μL) | Thermal cycling conditions |
| --- | --- | --- |
| Hieff UNICON Advanced qPCR SYBR Master Mix | 10 | (1) 95 ℃, 2 min; (2) 95 ℃, 10 s; (3) 60 ℃, 30 s; (4) Go to (2) for 39 cycles; (5) Melting curve |
| forward primer | 1 |  |
| reverse primer | 1 |  |
| cDNA (diluted fivefold) | 5 |  |
| nuclease-free water | 3 |  |
| Total | 20 |  |

**2. CCD Experiment**

As shown in **Table S7**, 20 experimental runs were conducted with different concentration combinations based on three significant factors identified from the screening step. (FeSO_4_·7H_2_O (A), MgSO_4_·7H_2_O (B), and maltose (C)). The highest yield of Ppd A reached 262.70 ± 10.92 mg/L.

**Table S7. CCD Design scheme and statistical analysis results**

| Number | A  （g/L） | B  （g/L） | C  （g/L） | Ppd A production  （mg/L） |
| --- | --- | --- | --- | --- |
| 1 | 0.005 | 0.2 | 30 | 231.28 ± 16.93 |
| 2 | 0.015 | 0.2 | 30 | 178.91 ± 20.41 |
| 3 | 0.005 | 0.6 | 30 | 262.70 ± 10.92 |
| 4 | 0.015 | 0.6 | 30 | 221.03 ± 26.26 |
| 5 | 0.005 | 0.2 | 40 | 254.17 ± 19.53 |
| 6 | 0.015 | 0.2 | 40 | 228.73 ± 24.02 |
| 7 | 0.005 | 0.6 | 40 | 255.55 ± 32.72 |
| 8 | 0.015 | 0.6 | 40 | 238.62 ± 41.81 |
| 9 | 0.0016 | 0.4 | 35 | 239.94 ± 21.82 |
| 10 | 0.0184 | 0.4 | 35 | 199.78 ± 23.41 |
| 11 | 0.01 | 0.064 | 35 | 184.36 ± 29.76 |
| 12 | 0.01 | 0.736 | 35 | 228.36 ± 10.98 |
| 13 | 0.01 | 0.4 | 26.6 | 217.78 ± 10.44 |
| 14 | 0.01 | 0.4 | 43.4 | 258.69 ± 10.33 |
| 15 | 0.01 | 0.4 | 35 | 233.98 ± 10.36 |
| 16 | 0.01 | 0.4 | 35 | 239.68 ± 14.91 |
| 17 | 0.01 | 0.4 | 35 | 245.70 ± 31.65 |
| 18 | 0.01 | 0.4 | 35 | 254.58 ± 14.35 |
| 19 | 0.01 | 0.4 | 35 | 250.84 ± 10.81 |
| 20 | 0.01 | 0.4 | 35 | 235.17 ± 25.53 |

**Table S8. ANOVA and model significance evaluation for CCD experiments**

| Source | Sum of Squares | df | Mean Square | *F*-value | *p*-value |
| --- | --- | --- | --- | --- | --- |
| Model | 9314.99 | 9 | 1035.00 | 9.63 | 0.0007 |
| A | 3045.60 | 1 | 3045.60 | 28.33 | 0.0003 |
| B | 1847.75 | 1 | 1847.75 | 17.19 | 0.0020 |
| C | 1690.96 | 1 | 1690.96 | 15.73 | 0.0027 |
| AB | 46.14 | 1 | 46.14 | 0.4292 | 0.5272 |
| AC | 333.65 | 1 | 333.65 | 3.10 | 0.1086 |
| BC | 484.65 | 1 | 484.65 | 4.51 | 0.0597 |
| A² | 420.66 | 1 | 420.66 | 3.91 | 0.0761 |
| B² | 1493.50 | 1 | 1493.50 | 13.89 | 0.0039 |
| C² | 17.32 | 1 | 17.32 | 0.1611 | 0.6966 |
| Residual | 1075.05 | 10 | 107.51 |  |  |
| Lack of Fit | 719.18 | 5 | 143.84 | 2.02 | 0.2292 |
| Pure Error | 355.88 | 5 | 71.18 |  |  |
| Cor Total | 10390.04 | 19 |  |  |  |

Based on the analysis of variance (ANOVA) results, a fitted equation was established to describe the relationship between the yield of Ppd A and the three factors:

$$\text{Y=242.88-14.93A+11.63B+11.13C+2.40AB+6.46AC-7.78BC-5.40}\text{A}^{\text{2}}\text{-10.18}\text{B}^{\text{2}}\text{+}$$

$\text{1.10}\text{C}^{\text{2}}$

(S7)

**References**

[1] Lehr F, Millies M, Mewes D. Bubble-Size Distributions and Flow Fields inBubble Columns[J]. AICHE Journal. 2004, 48, 2426-2443. https://doi.org/10.1002/aic.690481103

[2] Li Z J, Shukla V, Wenger K. Estimation of Hyphal Tensile Strengthin Production-Scale *Aspergillus oryzae* Fungal Fermentations[J]. Biotechnology and Bioengineering. 2002, 77, 601-613. https://doi.org/10.1002/bit.10209
